# An Ingenol Derived from *Euphorbia kansui* Induces Hepatocyte Cytotoxicity by Triggering G0/G1 Cell Cycle Arrest and Regulating the Mitochondrial Apoptosis Pathway *in Vitro*

**DOI:** 10.3390/molecules21060813

**Published:** 2016-06-22

**Authors:** Xiaojing Yan, Li Zhang, Yudan Cao, Weifeng Yao, Yuping Tang, Anwei Ding

**Affiliations:** 1 Jiangsu Collaborative Innovation Center of Chinese Medicinal Resources Industrialization, Jiangsu Key Laboratory for High Technology Research of TCM Formulae, Nanjing University of Chinese Medicine, Nanjing 210023, China; yanxiaojing963@163.com (X.Y.); raindc@163.com (Y.C.); njweifengyao@163.com (W.Y.); awding105@163.com (A.D.); 2 Changzhou Affiliated Hospital of Nanjing University of Chinese Medicine, Changzhou 213003, China

**Keywords:** lingenol, hepatotoxicity, cell cycle arrest, cell apoptosis, high content screen, ROS, Bcl-2/Bax

## Abstract

Natural product lingenol, a purified diterpenoid compound derived from the root of *Euphorbia kansui*, exerts serious hepatotoxicity; however, the molecular mechanisms remain to be defined. In the present study, cell counting Kit-8 (CCK-8), inverted phase contrast microscope and flow cytometry were used to demonstrate that lingenol significantly inhibited L-O2 cells proliferation, and induced cell cycle arrest and apoptosis. Moreover, the results investigated that lingenol markedly disrupted mitochondrial functions by high content screening (HCS). In addition, the up-regulation of cytochrome *c*, AIF and Apaf-1 and activation of caspases were found in L-O2 cells detected by Western blotting and ELISA assay, which was required for lingenol activation of cytochrome *c*-mediated caspase cascades and AIF-mediated DNA damage. Mechanistic investigations revealed that lingenol significantly down-regulated the Bcl-2/Bax ratio and enhanced the reactive oxygen species (ROS) in L-O2 cells. These data collectively indicated that lingenol modulation of ROS and Bcl-2/Bax ratio led to cell cycle arrest and mitochondrial-mediated apoptosis in L-O2 cells *in vitro*. All of these results will be helpful to reveal the hepatotoxicity mechanism of *Euphorbia kansui* and to effectively guide safer and better clinical application of this herb.

## 1. Introduction

The dried root of *Euphorbia kansui* T. N. Liou ex T. P. Wang (called kansui) is an effective and commonly used treatment of edema, ascite, and asthma [1]. Recent studies demonstrated that kansui has a wide range of pharmacological activities including tumor inhibition [2], anti-viral effects [3,4], regulation of immune system [5], modulatory effects on INF-γ [6], and treatment of diabetes [7]. However, kansui has highly toxic side effects in liver [8,9], which seriously restricts its clinical application.

**Figure 1:**
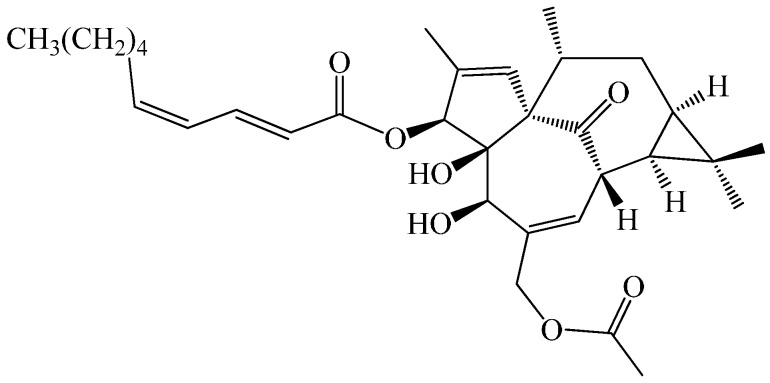
Chemical structure of 3EZ, 20Ac-ingenol.

Several terpenoids and phenolic derivatives have been isolated from kansui and identified in previous phytochemical investigations [10,11,12,13]. Terpenoids, a group of important bioactive compounds, are mainly in kansui, which exhibiting antiviral, anticancer and pesticidal effects [14,15]. Previously, our group investigated the mechanism that vinegar-processed kansui reduces kansui-induced hepatocyte cytotoxicity through decreasing the contents of toxic terpenoids and regulating the L-O2 cell apoptosis pathway [16]. Next, we successfully developed a bio-guided isolation method to separate 12 terpernoids from ethyl acetate (EtOAc) of kansui and simply investigated the hepatic cytotoxicity against L-O2 cell lines *via* 3-(4,5-dimethylthiazol-2-yl)-2,5-diphenyltetrazolium bromide (MTT) assay [17]. These results demonstrated 3-*O*-(2′*E*,4′*Z*-decadienoyl)-20-*O*-acetylingenol (3EZ, 20Ac-ingenol, Figure 1), a member of the terpernoids, exhibited strong inhibition of cell proliferation against human liver cell line L-O2 with low IC_50_ values of 12.40 μM, but the exact mechanism was not clearly represent.

Increasing studies have demonstrated that apoptosis is a key determinant of liver injury, which can usually be initiated by intrinsic (mitochondrial) and extrinsic (death receptors) pathways [18,19]. Mitochondria are the main source of reactive oxygen species (ROS) in the cell and involved in cell demise, and therefore, mitochondria-related apoptosis could be a major mechanism of drug-induced liver disease [20]. The intrinsic mitochondrial pathway involving Bcl-2 family proteins integrates extracellular information to promote the mitochondrial permeability, decrease the mitochondrial membrane potential, trigger depletion of outer membrane potential, release of proteins from mitochondrial into cytosol, and activation of the caspase family [21,22].

The current study investigated the hepatotoxicity mechanism of 3EZ, 20Ac-ingenol via regulating the mediators of mitochondrial apoptosis pathway in L-O2 cells. These results showed that 3EZ, 20Ac-ingenol could induce the hepatotoxicity of L-O2 cells by increasing the intracellular ROS and regulating the mediators of mitochondrial pathway hepatocyte apoptosis. In addition, cytochrome *c*-mediated caspase activation and apoptosis-inducing factor (AIF)-mediated DNA damage were found in 3EZ, 20Ac-ingenol-treated L-O2 cells. Furthermore, mechanistic investigations revealed that these effects were mediated by the ROS and Bcl-2/Bax ratio. This research is to lay the foundation for safer and better clinical application of kansui.

## 2. Results

### 2.1. Effects of 3EZ, 20Ac-Ingenol on L-O2 Cell Viability and Cell Morphology

**Figure 2:**
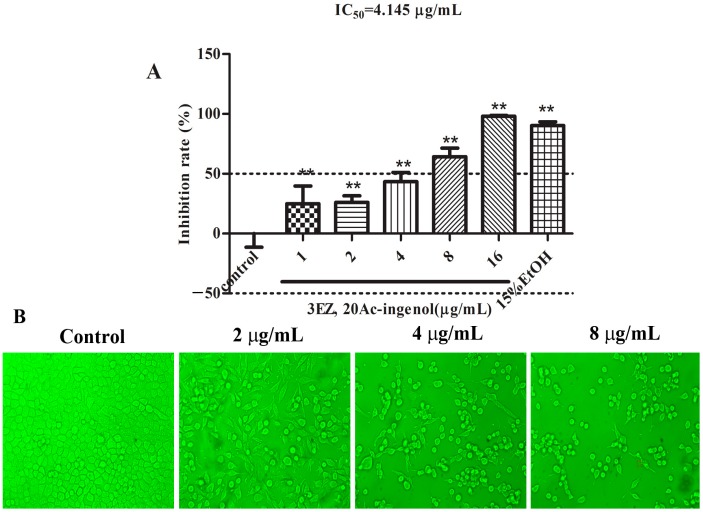
The effects of 3EZ, 20Ac-ingenol on hepatocyte L-O2 cells viability (×200). (**A**) The cell inhibition rate of 3EZ, 20Ac-ingenol using CCK-8 assay. Results are shown as mean ± SD (*n* = 6), ** *P* < 0.01 compared with control group. (**B**) Cellular morphologic changes were observed under inverted phase contrast microscope. L-O2 cells were treated with 3EZ, 20Ac-ingenol at the concentration of 2, 4 and 8 μg/mL for 48 h, respectively.

To investigate whether 3EZ, 20Ac-ingenol has toxic effects on L-O2 cells, a wide range of doses for 3EZ, 20Ac-ingenol from 1 to 16 μg/mL were incubated with L-O2 cells for 48 h. CCK-8 assay was used to determined cell viability. As shown in Figure 2A, compared with control group, 3EZ, 20Ac-ingenol significantly increased the inhibition rate of L-O2 cells in a dose-dependent manner (*P* < 0.01), and the IC_50_ value of 3EZ, 20Ac-ingenol was 4.145 μg/mL. Moreover, L-O2 cells in control group were well-adhered and normal morphology detected by inverted phase contrast microscopy, while cells treated with 3EZ, 20Ac-ingenol for 48 h, had remarkable morphological changes in a dose-dependent manner, becoming irregular and shrunken compared with control group (Figure 2B).

### 2.2. Effects of 3EZ, 20Ac-Ingenol on L-O2 Cell Cycle and Apoptosis

**Figure 3:**
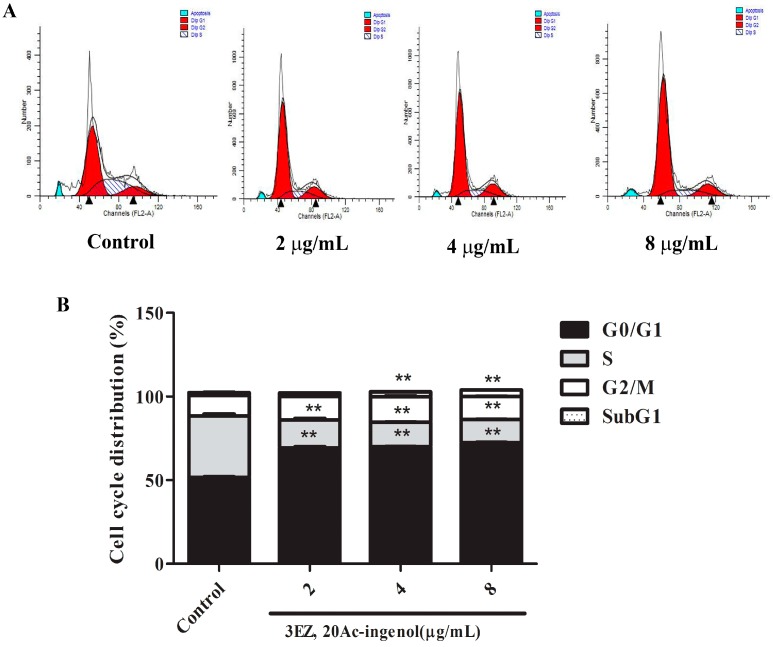
The effects of 3EZ, 20Ac-ingenol on L-O2 cell cycle using flow cytometer: (**A**) cell cycle distribution; and (**B**) cell number percentage in each phase (subG1, G0/G1, S and G2/M) were detected and calculated. Quantification of apoptotic cells were analyzed and expressed. Data are presented as mean ± SD from triplicate samples, ** *P* < 0.01 compared with control group.

**Figure 4:**
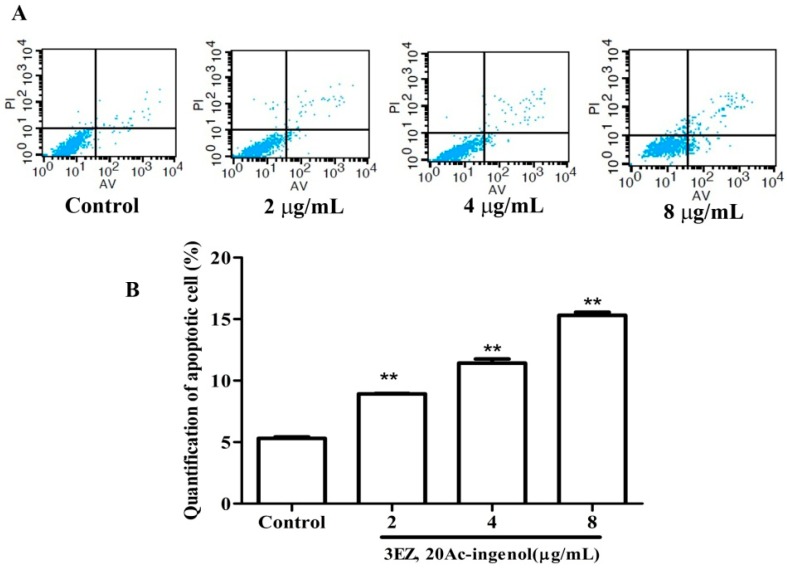
The effects of 3EZ, 20Ac-ingenol on L-O2 cell apoptosis using flow cytometer: (**A**) Images; and (**B**) Quantification of apoptotic cells were analyzed and expressed. Data are presented as mean ± SD from triplicate samples, ** *P* < 0.01 compared with control group.

Flow cytometric analysis of L-O2 cells stained with PI showed a mild increase (*P* < 0.01) in G0/G1 and SubG1 phase and an obvious decrease (*P* < 0.01) in S phase after the cells were treated with the different concentrations of 3EZ, 20Ac-ingenol for 48 h (Figure 3A,B). Our results demonstrated that 3EZ, 20Ac-ingenol could arrest L-O2 cells at G0/G1 phase and induce L-O2 apoptosis. To gain further insight into the L-O2 cell apoptosis, Annexin V-FITC was used to quantitatively determine the percentage of cells that were actively undergoing apoptosis. As shown in Figure 4A,B, in 3EZ, 20Ac-ingenol group, the apoptotic cells were significantly increased (*P* < 0.01) in a dose-dependent manner compared with control group. Collectively, 3EZ, 20Ac-ingenol could induce L-O2 cell apoptosis.

### 2.3. Effects of 3EZ, 20Ac-Ingenol on the Generation of Reactive Oxygen Species (ROS) 

**Figure 5:**
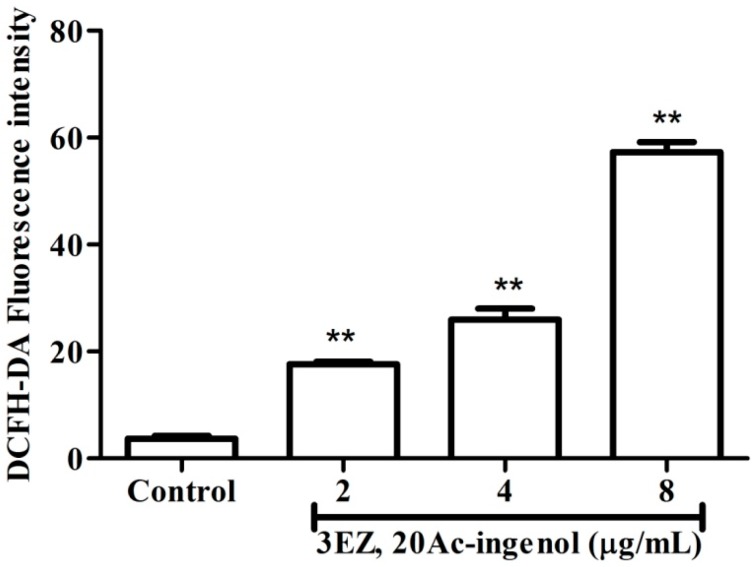
The effects of 3EZ, 20Ac-ingenol on ROS generationin L-O2 cells using DCFH-DA staining by laser scanning confocal microscope. Quantification of intracellular ROS fluorescence intensity were analyzed and expressed. Data are presented as mean ± SD from triplicate samples, ** *P* < 0.01 compared with control group.

It has been confirmed that ROS generation plays an important role in pro-apoptotic activities, and excess ROS promotes cell death [23,24]. To assess whether oxidative stress damage was involved in 3EZ, 20Ac-ingenol-induced apoptosis, L-O2 cells were exposed with 3EZ, 20Ac-ingenol following detection of intracellular ROS level using DCFH-DA staining by laser scanning confocal microscope. As shown in Figure 5, the intracellular ROS level of L-O2 cells was significantly increased in 3EZ, 20Ac-ingenol-treated cells in a dose-dependent manner.

### 2.4. Effects of 3EZ, 20Ac-Ingenol on Mitochondrial Function

**Figure 6:**
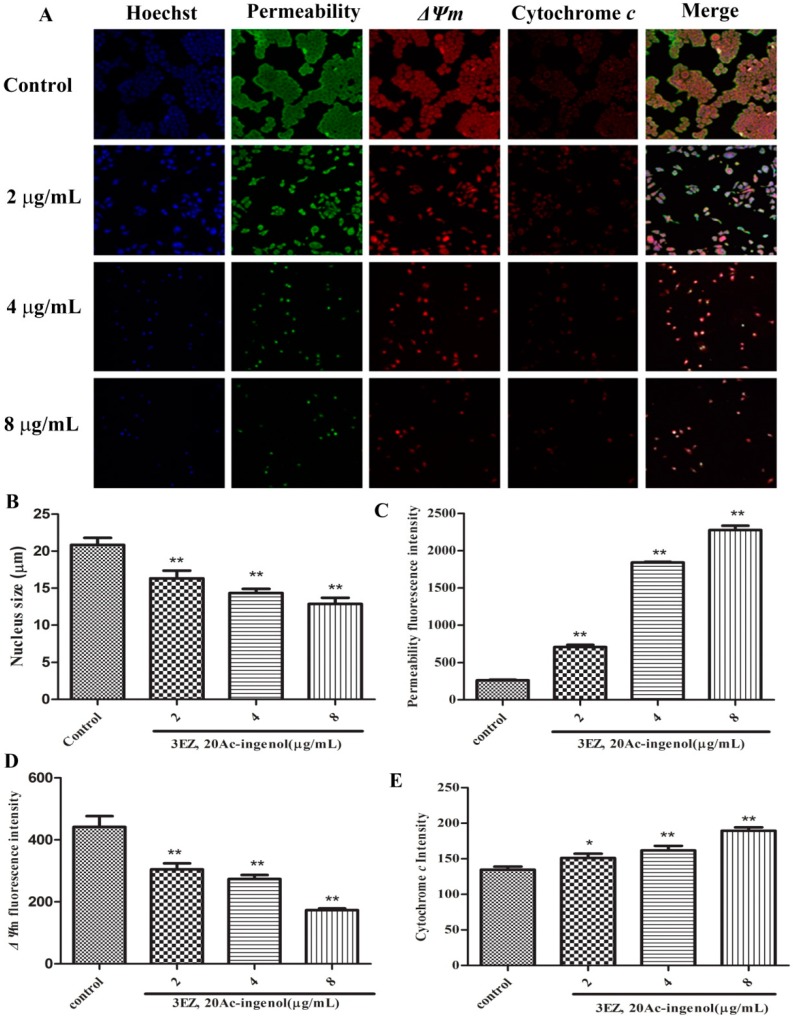
The mitochondrial function induced by 3EZ, 20Ac-ingenol in apoptotic hepatocyte L-O2 cells (×100). (**A**) Images of cells were taken by the high content screening (HCS) Assay Scan VTI Reader. (**B**) The average cell nuclear size was stained by Hoechst 33342 and quantified by the HCS Assay Scan software. (**C**) The cell membrane permeability was manifested by the average intensity of fluorescent dyes permeabilized through cell membranes. (**D**) The cell mitochondrial membrane potential (*ΔΨ*m) was manifested by the average intensity of mitochondrial membrane potential fluorescent dyes. (**E**) The release of cytochrome *c* from mitochondria was manifested by the average cytochrome *c* intensity of fluorescent dye. Data are presented as mean ± SD from triplicate samples, * *P* < 0.05, ** *P* < 0.01 compared with control group.

**Figure 7:**
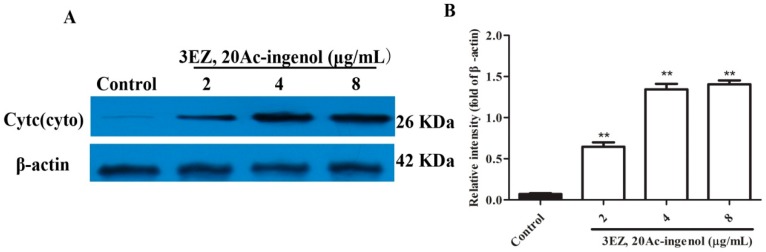
The effects of 3EZ, 20Ac-ingenol on the expression of cytc (cyto). L-O2 cells were incubated with 2, 4 and 8 μg/mL of 3EZ, 20Ac-ingenol for 48 h, and then cytc (cyto) was assessed via Western blotting (**A**). Quantification of cytc (cyto) was analyzed and expressed (**B**). Values are expressed as mean ± SD from three independent experiments, ** *P* < 0.01 compared with control group.

To further elucidate the mechanism of cell apoptosis, the cellular morphological changes were evaluated and the specific mechanism by which 3EZ, 20Ac-ingenol induces hepatocyte apoptosis using high content screening (HCS) analysis was explored. Nuclear, cell membrane permeability transition (MPT), mitochondrial membrane potential (*ΔΨ*m) and the release of cytochrome *c* from mitochondrial were co-stained with fluorescent dyes, and then images were taken with an HCS assay scan VTI Reader. Data derived from these images were analyzed by HCS software. The results (Figure 6) suggested that mitochondrial pathway is involved in 3EZ, 20Ac-ingenol-induced hepatocyte apoptosis. Compared with control group, cells were exposed to 3EZ, 20Ac-ingenol for 48 h, the nucleus size and *ΔΨ*m fluorescent intensity significantly decreased (*P* < 0.01) and MPT and the release of cytochrome *c* from mitochondrial fluorescent intensity markedly increased (*P* < 0.05) (Figure 6B–E). Besides, Western blotting was conducted to detect the cytochrome *c* in cytosolic fractions (Figure 7A,B), Based on the results, the expression of cytochrome *c* significantly increased (*P* < 0.01) in cytosolic fraction.

### 2.5. Effects of 3EZ, 20Ac-Ingenol on Caspases Activation

**Figure 8:**
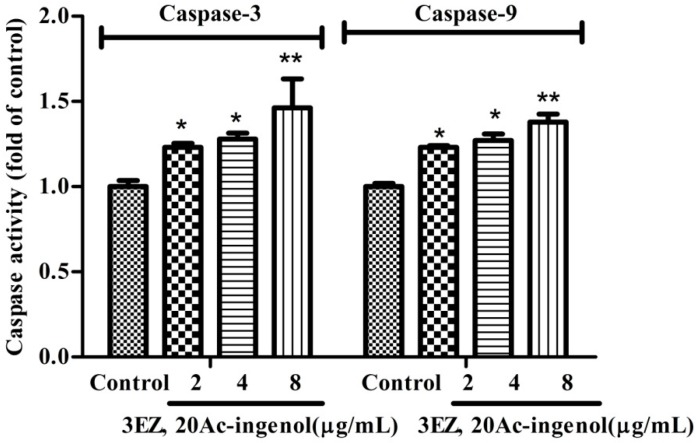
The effects of 3EZ, 20Ac-ingenol on caspases activation. L-O2 cells were treated with 3EZ, 20Ac-ingenol at the concentration of 2, 4, 8 μg/mL for 48 h, respectively. The caspases activation was assayed by ELISA assay. Values are expressed as mean ± SD from three independent experiments, * *P* < 0.05, ** *P* < 0.01 compared with control group.

Subsequently, despite varying conditions under which apoptosis can occur, caspase activation is a universal event, and, for that reason, is considered an apoptotic marker. In the current study, as detected by ELISA assay, this study showed that the caspase-3 and caspase-9 (*P* < 0.05) activities were markedly increased in 3EZ, 20Ac-ingenol group compared with control group (Figure 8).

### 2.6. Effects of 3EZ, 20Ac-Ingenol on Mitochondrial Apoptotic Proteins

**Figure 9:**
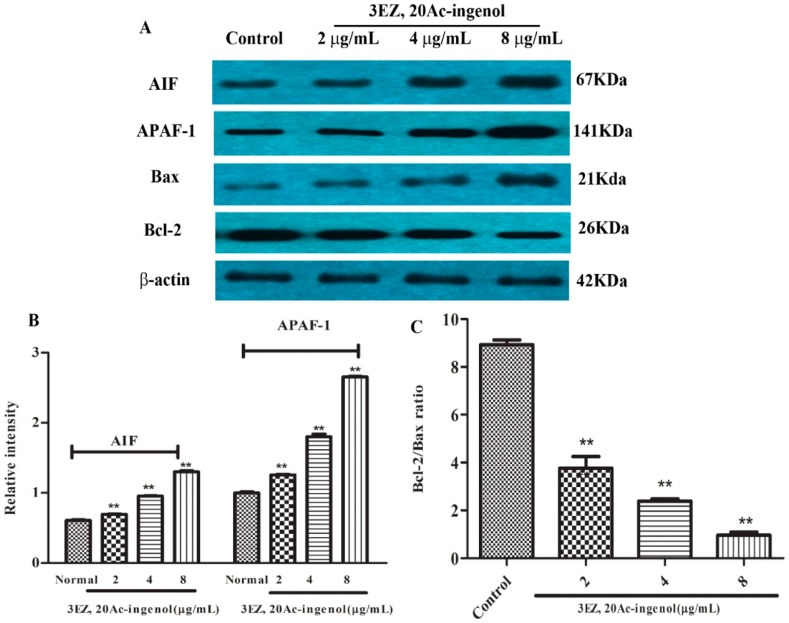
The effects of 3EZ, 20Ac-ingenol on the expression of AIF, APAF-1 and Bax/Bcl-2 protein ratio. L-O2 cells were incubated with 2, 4 and 8 μg/mL of 3EZ, 20Ac-ingenol for 48 h, and then Bax, Bcl-2, AIF, APAF-1 and β-actin were assessed via Western blotting (**A**). Quantification of AIF and APAF-1 (**B**) and Bcl-2/Bax ratio (**C**) were analyzed and expressed. Values are expressed as mean ± SD from three independent experiments, ** *P* < 0.01 compared with control group.

The molecular mechanism by which 3EZ, 20Ac-ingenol induced L-O2 cell apoptosis was investigated. Apoptosis-inducing factor (AIF) is essential for programmed cell death in early morphogenesis during embryogenesis [25]. Many kinds of cytotoxic injuries that induce AIF translocation to the nucleus have been reported [26,27,28]. The present study showed that 3EZ, 20Ac-ingenol could significantly increase AIF expression (*P* < 0.01) (Figure 9A,B). Apoptotic protease-activating factor 1 (APAF-1) is one of the key regulators in the mitochondrial apoptotic pathway. APAF-1 binds to a protein called cytochrome *c*, which is released from mitochondria and this complex activates caspase-9, which then triggers executioner caspases, leading to apoptosis [29]. This study showed that 3EZ, 20Ac-ingenol apparently increases the protein of APAF-1 (*P* < 0.01) (Figure 9A,B), suggesting that this regulator was involved in 3EZ, 20Ac-ingenol effects. Alternatively, this study turned to Bcl-2/Bax ratio that control apoptotic signal transduction. Since the Bcl-2/Bax ratio is critical for regulating the release of cytocrome *c* from mitochondria, the potential involvement of the Bcl-2 family proteins in the process of 3EZ, 20Ac-ingenol mediated hepatocyte apoptosis was investigated. As shown in Figure 9A,C, compared with control group, 3EZ, 20Ac-ingenol-treated cells showed an obviously decreased Bcl-2/Bax protein ratio (*P* < 0.01). Collectively, these data indicated that 3EZ, 20Ac-ingenol could activate AIF and APAF-1 expression and down-regulate Bcl-2/Bax ratio leading to mitochondrial-dependent pathway apoptosis in L-O2 cells.

## 3. Discussions 

As a toxic Chinese medicinal herb, kansui has to be stir-baked with vinegar to reduce its toxicity for clinic application. Recent studies have shown that kansui could induce the hepatocyte toxicity [16,17]. However, the potential hepatotoxicity mechanisms of kansui are still not fully understood. 3EZ, 20Ac-ingenol, is a natural cytotoxic diterpenoid extracted from kansui, which exhibits a strong cytotoxic activity in human normal cell line L-O2 [17]. Therefore, this study was carried out to indicate 3EZ, 20Ac-ingenol′s specific hepatotoxic mechanism.

Initially, this study examined the cell viability of 3EZ, 20Ac-ingenol. The results obtained by CCK-8 assay and inverted phase contrast microscope demonstrated that 3EZ, 20Ac-ingenol markedly inhibited L-O2 cell proliferation, which indicates that it has obvious hepatotoxicity. As an important mechanism for cell death, apoptosis is well known to be involved in the process of hepatocyte damage [30]. Hepatocyte apoptosis was found in many physiological processes and can contribute to the development of many liver diseases [31,32]. The subsequent cell cycle analysis indicated that 3EZ, 20Ac-ingenol arrested cell cycle at the G0/G1 transition and induced cell apoptosis. Measured using Annxin V-FITC/PI Kit, the results supported that 3EZ, 20Ac-ingenol could induce L-O2 cell apoptosis.

Apoptosis can be initiated via two alternative signaling pathways: the death receptor-mediated extrinsic apoptotic pathway and the mitochondrial-mediated intrinsic apoptotic pathway [18]. Mitochondrial dysfunction such as the change of the mitochondrial membrane permeability often accompanies the release of several mitochondrial proteins into the cytoplasm, which appears to be important for progression of the apoptotic pathway [20]. Upon induction of apoptosis, activation of this ion channel complex triggers mitochondrial membrane permeabilization that finally results in an increased permeability of the outer mitochondrial membrane, which permits the release of two main groups of mitochondrial pro-apoptotic proteins, which including the caspase-dependent mitochondrial way of apoptosis (cytochrome *c*) and caspase-independent mitochondrial way of apoptosis (AIF) [33,34]. Next, using HCS analysis, the effect of 3EZ, 20Ac-ingenol on cell nuclear size, MPT, *ΔΨ*m and the release of cytochrome *c* from mitochondria were assessed. Strikingly, results confirmed that 3EZ, 20Ac-ingenol could induce hepatocyte apoptosis via stimulating the mitochondria, increasing MPT, reducing *ΔΨ*m and then leading to the release of cytochrome *c* from the inter-membrane space.

A further characteristic of apoptosis is the activation of caspase family members. It is now well established that, in most cell types, once cytochrome *c* is released into the cytosol, it triggers caspase-9 activation, results in the cleavage of caspase-3, and ultimately leads to the activation of the execution phase of apoptosis [35]. In this study, data demonstrated that 3EZ, 20Ac-ingenol significantly increased the caspase-3 and caspase-9 activation. These observations suggested that 3EZ, 20Ac-ingenol induced L-O2 cell apoptosis by activating caspase-3 and caspase-9, which could be due to upstream events in the intrinsic pathway.

AIF exhibits a unique pathway in causing apoptosis independent of caspase activation [36], which located in the mitochondrial inter-membrane space and induced translation from the mitochondria to the cytosol and nucleus [37]. APAf-1-mediated apoptosome formation could direct damage to mitochondria, which results in cytochrome *c* release and caspase-9 activation [38,39]. This study uncovered that AIF and APAF-1 expression in L-O2 cells were up-regulated by 3EZ, 20Ac-ingenol. Briefly, both cytochrome *c*-APAF-1-mediated caspase activation and AIF-mediated caspase-independent mitochondrial way were responsible for the mechanism of 3EZ, 20Ac-ingenol-induced mitochondrial apoptosis in L-O2 cells.

Mitochondrial apoptosis pathway can be initiated by intracellular stimuli and mediated by the Bcl-2 family proteins that act as sensors to integrate death and survival signals [40]. The ratio of Bcl-2/Bax is a pivotal determinant and its reduction leads to increase of mitochondrial outer membrane permeabilization and release of cytochrome *c*, and then finally triggers activation of caspase cascades culminating in cellular fragmentation [41]. To determine which apoptosis-related proteins are regulated by 3EZ, 20Ac-ingenol, the expression of Bax and Bcl-2 protein were measured using Western blotting analysis. In this study, data showed that 3EZ, 20Ac-ingenol down-regulated Bcl-2/Bax protein ratio in L-O2 cells, thus, suggesting that the 3EZ, 20Ac-ingenol initiated L-O2 cells to undergo intrinsic apoptosis dependent on down-regulation of Bcl-2/Bax protein ratio, which could induce both the cytochrome *c*-mediated the caspase-dependent and AIF-mediated the caspase-independent mitochondrial apoptosis. 

**Figure 10:**
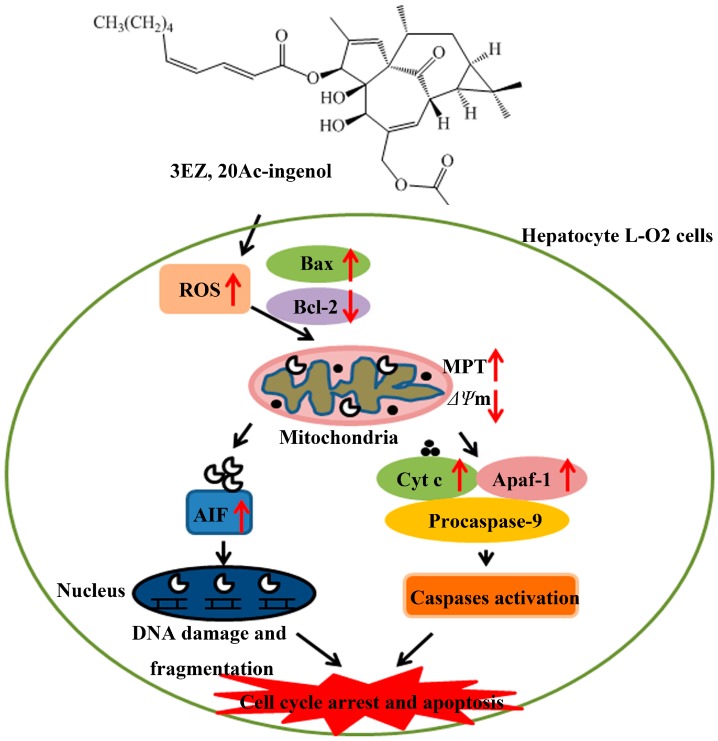
Proposed mechanisms of 3EZ, 20Ac-ingenol-induced cell apoptosis in hepatocyte L-O2 cells. 3EZ, 20Ac-ingenol may directly activation of ROS, and then down-regulate Bcl-2/Bax ratio, increase the MPT and decrease the *ΔΨ*m leading to the release of proapoptotic proteins, such as cytochrome *c* and AIF. Next, a group of cytochrome *c* was mediated by APAf-1 to apoptosome formation and consequently activating caspase-9 activity, further, activating caspase-3 and inducing cell apoptosis. The other group of AIF located in the mitochondrial inter-membrane space and induced translation from the mitochondria to the cytosol and nucleus, and then cleaved DNA, which finally induced cell cycle arrest and caspase-independent mitochondrial pathway apoptosis.

Oxidative stress caused by ROS controls the expression of Bcl-2 family proteins [42] and plays a key role in the induction of apoptosis [43,44]. Excessive ROS destroyed the mitochondrial membrane integrity, leading to cytochrome c and AIF release, caspase activation and finally apoptosis [45]. The present study demonstrated that 3EZ, 20Ac-ingenol could elevate ROS, regulate the expression of Bcl-2/Bax, promote mitochondrial dysfunction, release of cytochrome *c* and AIF and finally activate the caspases. Taken together, based on our observations, a simplified mode was proposed in Figure 10 to describe the mechanisms of 3EZ, 20Ac-ingenol induction of cell mitochondrial apoptosis in hepatocyte L-O2 cells. Additional studies are ongoing in our laboratory to validate the present findings *in vivo.*

## 4. Experimental Section 

### 4.1. Plant Materials

The root of *Euphorbia kansui* T. N. Liou ex T. P. Wang was collected from Red River valley of Baoji (China), and identified by Professor Chungen Wang (Nanjing University of Chinese Medicine, Nanjing, China). The voucher specimen was deposited in the Herbarium of Nanjing University of Chinese Medicine, Nanjing, China.

### 4.2. Preparation of Sample Solutions

3-*O*-(2′*E*,4′*Z*-decadienoyl)-20-*O*-acetylingenol (3EZ, 20Ac-ingenol) was isolated from the EtOAc extract of kansui as described previously and the purity is above 98% [17]. By spectral and physiochemical data analysis and/or comparison with literatures data, their structures were elucidated as 3-*O*-(2′*E*,4′*Z*-decadienoyl)-20-*O*-acetylingenol. 3EZ, 20Ac-ingenol was carefully weighed 5 mg, dissolved in DMSO as a 5 mg/mL stock solution, respectively, and subsequently diluted into 1, 2, 4, 8 and 16 μg/mL of serial solutions for further cytotoxicity studies.

### 4.3. Chemical and Reagents

Dulbecco′s modified Eagle′s medium (DMEM) was purchased from Gibco (Grand Island, NY, USA). Fetal Bovine Serum (FBS) was obtained from Sijiqing Biotechnology Co., Ltd., (Hangzhou, China). Penicillin and streptomycin were purchased from HyClone, Thermo Fisher Scientific Inc., (Waltham, USA). Cell Counting Kit-8 (CCK-8) was purchased from Dojindo Laboratories Science and Technology, Inc., (Kumamoto, Japan). Dimethylsulfoxide (DMSO) was obtained from Beijing Solarbio Science and Technology Co., Ltd., (Beijing, China). Propidium iodide (PI) was purchased from Sigma-Aldrich, Sigma Chemical Co., (St. Louis, MO, USA). Multiparameter Cytotoxicity 3 Kit was purchased from Thermo Fisher Scientific Inc. Anti-cytc, Anti-Bax, anti-Bcl-2, anti-AIF, anti-Apaf-1 and β-actin were purchased from Santa Cruz Biotechnology, Inc. (Santa Cruz, CA, USA). Annxin V-FITC/PI double staining Kit, caspase-3 and caspase-9 ELISA Kits were obtained from Nanjing KeyGen Biotech. Co., Ltd., (Nanjing, China).

### 4.4. Cell Line and Cell Culture

Human Normal Liver cells L-O2 was purchased from Shanghai Institute of Biochemistry and Cell Biology. Cells were cultured in Dulbecco’s modified Eagle′s medium (DMEM) (Gibco, Grand Island, NY, USA) and supplemented with 10% heat-inactivated FBS (Sijiqing Biotechnology Co. Hangzhou, China) and 100 unit/mL penicillin, and 100 μg/mL streptomycin at 37 °C with a humidified atmosphere of 5% CO_2_.

### 4.5. Cell Viability Analysis

Cytotoxicity activity was assayed by Cell Counting Kit-8 (CCK-8) (Dojindo Laboratories Science and Technology, Inc., Kumamoto, Japan) as described [46,47]. Briefly, cells were seeded into 96-well plates (Costar, Corning Inc., Corning, New York, USA) at density of 1 × 10^4^ cells/well, cultured for 24 h, and then divided into control group dimethylsulfoxide (DMSO), 3EZ, 20Ac-ingenol group (the final sample solutions at five concentration of 1, 2, 4, 8, 16 μg/mL and 15% EtOH group. After incubation for 48 h, the cell viability was assayed by CCK-8. All experiments were performed at least three times. Data were calculated as the percentage of cell:

Inhibition Rate (%) = (1 – sample solution absorbance value/control absorbance value) × 100%.
(1)

### 4.6. Cell Morphological Detection

Cells were seeded in a 6-well plate at a concentration of 1.0 × 10^5^ cells/mL, and incubated for 24 h at 37 °C in a humidified atmosphere with 5% CO_2_, and then were treated with 3EZ, 20Ac-ingenol at various concentration of 2, 4, 8 μg/mL. After incubation for 48 h, the morphology of L-O2 cells was observed under an inverted phase contrast microscope (Olympus, Tokyo, Japan).

### 4.7. Cell Cycle Analysis

To determine cell cycle distribution, Propidium Iodide (PI) cell cycle Kit was performed to detect by flow cytometer [48]. L-O2 cells were seeded in 6-well plates at a density of 1 × 10^4^ cells/well and incubated for 24 h, and then exposed to 3EZ, 20Ac-ingenol at the concentration of 2, 4, 8 μg/mL, respectively. After treatment for 48 h, the cells were suspended in a fluorochrome solution containing 5 μg/mL PI, 1 mg/mL sodium citrate, 0.1 mg/mL RNase and 1% Triton X-100, and then analyzed with a flow cytometer (FACSCalibur, BD Instruments Inc., San Jose, USA) and FlowJo 7.1.0 software (Ashland, OR, USA). All experiments were performed in triplicate, and for each measurement, at least 20,000 cells were counted.

### 4.8. Cell Apoptosis Analysis

Apoptotic cell death was measured by Annexin V-FITC and PI double staining followed by flow cytometry [49,50]. After L-O2 cells were exposed to 3EZ, 20Ac-ingenol at the concentration of 2, 4, 8 μg/mL for 48 h in 6-well plates, cells were staining with Annxin V-FITC/PI Kit according to the manufacturer′s instructions and then analyzed with a flow cytometer. All experiments were performed in triplicate, and for each measurement, at least 20,000 cells were counted. 

### 4.9. Intracellular Reactive Oxygen Species (ROS) Detection

Intracellular ROS production was assessed by 2′,7′-dichlorfluorescein diacetate (DCFH-DA) staining [51]. Briefly, L-O2 cells were plated into a 6 well plate at 1 × 10^5^ cells/well for 24 h, and then exposed with 3EZ, 20Ac-ingenol at various concentration of 2, 4, 8 μg/mL. After 48 h incubation, cells were washed with PBS for three times and subsequently stained with DCFH-DA (10 μm) at 37 °C for 30 min. Then cells were washed with PBS for three times and resuspended in PBS. The fluorescence of ROS generation was detected by laser scanning confocal microscope (excitation at 488 nm; emission at 525 nm).

### 4.10. High Content Screening (HCS) Analysis of Mitochondrial Function

Using HCS technology, the 3EZ, 20Ac-ingenol at different doses inducing L-O2 mitochondrial pathway apoptotic cells were assayed by Assay Scan VTI HCS Reader with the Cellomics^®^ Multiparameter Cytotoxicity 3 Kit (Thermo Fisher Scientific Inc., Waltham, Massachusetts, USA). The principle of the assay was that cells were labeled with flurorescent dyes that would indicate the cellular properties of interest, including nucleus, MPT and *ΔΨ*m and cytochrome *c*. All procedures were performed according to the manufacturer′s instructions [16,52]. Afterwards, plates were sealed and run immediately on the Assay Scan VTI HCS Reader to acquire images. Images were analyzed with HCS software (Thermo Fisher Scientific Inc., Waltham, Massachusetts, USA).

### 4.11. Elisa Assay

The measurement of caspase-3 and caspase-9 activities was performed according to the manufacturer′s instructions [53]. Briefly, the treated cells were washed twice with ice-cold PBS and then were used for caspase-3 and caspase-9 activities assay, respectively (Nanjing Jiancheng Bioengineering Institute, Nanjing, China). The activity of caspase-3 and caspase-9 were quantified spectrophotometrically at 405 nm using ELISA reader (Nanjing, China). 

### 4.12. Western Blot Analysis

Protein were resolved on SDS-PAGE and detected as described previously [54,55]. The following antibodies were used in our studies: anti-cytc (cyto), anti-Bax, anti-Bcl-2, anti-AIF, anti-Apaf-1 and β-actin were purchased from Santa Cruz Biotechnology, Inc. (Santa Cruz, CA, USA).

### 4.13. Statistical Analysis

All experimental values were presented as mean ± SD. The results were analyzed using one-way *ANOVA* followed by *LSD* tests, and Students’ *t*-test was used for the two groups′ comparison as needed. Values of *P* < 0.05 were considered to be statistically significant.

## 5. Conclusions

In summary, the above results confirmedly demonstrated the 3EZ, 20Ac-ingenol has strong hepatotoxicity, which stimulated mitochondrial apoptosis dependent on cytochrome *c*-mediated caspase activation and AIF-mediated caspase-independent pathway in hepatocyte L-O2 cells. Molecular evidence indicated that promotion of ROS and down-regulation of Bcl-2/Bax ratio could be direct target molecules for 3EZ, 20Ac-ingenol within L-O2 cells. All of these results will be helpful to reveal the hepatotoxicity mechanism of kansui, and, further, to effectively guide safer and better clinical application of this herb.
